# Evaluation of Knowledge, Awareness, and Factors Associated with Diabetes: A Cross-Sectional Community-Based Study

**DOI:** 10.1155/2022/1921010

**Published:** 2022-07-16

**Authors:** Wajid Syed, Mohammad K. Alharbi, Osama A. Samarkandi, Ahmed Alsadoun, Mahmood Basil A. Al-Rawi, Ayesha Iqbal, Sana Samreen

**Affiliations:** ^1^Department of Clinical Pharmacy, College of Pharmacy, King Saud University, Riyadh 11451, Saudi Arabia; ^2^Department of Nursing Administration and Education, College of Nursing, King Saud University, Riyadh, Saudi Arabia; ^3^Nursing Informatics Vice Dean for Academic Affairs, Prince Sultan College for Emergency Medical Services, King Saud University, Riyadh 11466, Saudi Arabia; ^4^Department of Medical Surgical College of Nursing, King Saud University, Riyadh, Saudi Arabia; ^5^Department of Optometry, College of Applied Medical Sciences, King Saud University, Riyadh, Saudi Arabia; ^6^Division of Pharmacy Practice and Policy, School of Pharmacy, University Park Campus, University of Nottingham, Nottingham NG7 2RD, UK; ^7^Aurobindo College of Pharmacy, Warangal, Telangana, India

## Abstract

**Methods:**

A cross-sectional study was carried out for over 4 months from May to August 2020 using a self-administered, anonymous online questionnaire. All adults of both genders were invited to participate in the study. Individuals who are able to read and understand the English language were included in the study. Data were descriptively analyzed using a statistical package for social science version 26 (SPSS).

**Results:**

A total of 427 subjects responded to the questionnaires. More than half of them were male 253 (59.3%), while 174 (40.7%) were female, approximately 49% had a university degree, and 196 (45.9%) were postgraduates. The mean age of the participants was 25.7 ± 6.2 (mean ± SD). Most of them were single 230 (53.9%). The majority of the respondents 367 (85.9%) had heard of hyperglycemia, while a comparable number of 366 (85.7%) were aware of it being a lifelong disease. About 305 (71.4%) of participants knew that genetic factors were associated with hyperglycemia, and more than half of 250 (58.5%) knew that diabetes causes foot problems. There was a significant association between the cause of diabetes with income and educational status (*p* < 0.05) and diabetic complications with age, education, and family income (*p* < 0.05).

**Conclusion:**

In conclusion, there is a need for greater awareness and additional education regarding hyperglycemia among the general community in the state of Telangana, India, to reduce the incidence of diabetes and associated risk factors. Public health campaigns that encourage healthier lifestyles might help achieve this aim.

## 1. Introduction

Diabetes is a chronic disease, affecting people of both genders and across all age groups, and is currently considered a major public health challenge worldwide [1]. Globally, estimates suggest that nearly 425 million adults aged between 20 and 79 years are currently living with diabetes, and this number is expected to reach 629 million by 2045 [2]. According to the World Health Organization (WHO), approximately, 69.2 million people were living with diabetes in India in 2015, with this number expected to reach 98 million by 2030, making India a country with the highest diabetes incidence after China [2, 3]. In India, the prevalence of diabetes in the capital city of the Telangana state has been estimated at 16.6%, which is higher than for comparable cities, such as Mumbai (7.5%), Chennai (13.5%), and Bangalore (11.7%) is followed by the capital of India at 11.06% [4, 5]. More recently, a large community-based study from North India, using a sample of 5127 individuals aged between 25 and 44 years, reported an overall prevalence of diabetes at 8.3% [6].

Although raising awareness of risk factors and complications associated with diabetes has been a common strategy for controlling disease incidence. For example, Singla et al. measured diabetes awareness in a visiting outpatients in Dehradun over 6 months, revealing that 56% were aware of diabetes [7]. Similarly, a study by Muninarayana et al., assessing the prevalence and awareness of diabetes mellitus among 311 adults in rural areas of India, showed that 50% of participants were aware of diabetes [8]. Moreover, another study by Aljin et al., using a sample of 258 patients with type 2 diabetes in the Tamil Nadu state, showed that 63.1% of them had adequate knowledge regarding type 2 diabetes mellitus [9] However, a study by Mohan et al., assessing awareness and knowledge about diabetes among the general population, as well as among individuals with diabetes in 4 selected regions of India, revealed poor knowledge and awareness of diabetes, particularly in rural areas [10]. According to preliminary research, a gap or lack of knowledge of chronic diseases can increase morbidity and mortality rates [11]. Several studies, however, assessed knowledge, attitudes, and practice regarding other acute and chronic diseases such as malaria [12], intestinal parasitic infection [13], influenza [14], and corona virus [15]. Until now, there have been few studies that looked at community-wide knowledge and awareness in response to diabetes [16–18]. According to published studies, adequate knowledge and awareness and strict adherence and self-care measures would have a significant impact on morbidity and mortality rates, thereby aiding in disease control. To the best of our knowledge, there are currently limited data about knowledge and awareness of diabetes among the general population of the Telangana state, India. Thus, this study aimed to evaluate public knowledge and awareness regarding diabetes in the state of Telangana.

## 2. Methods

A cross-sectional study design was used to collect the data. An electronic survey was developed, using Google forms, and distributed among the general population in the state of Telangana, India, over 4 months, from May to August 2020 using social media as the platform to collect the data. Participants were recruited through the snowball technique (an individual who is recruited in the study will provide referrals). The study included individuals aged >18 years or more, of both genders, who can read and understand the English language, and are willing to provide informed consent. Others who do not match the inclusion criteria were excluded from the study. Data collection was completed using a structured, self-administered questionnaire.

To explore diabetes knowledge and awareness, we developed a questionnaire, following a thorough review of the relevant literature. The questionnaire was validated for content and ease of use, with the help of an advisor with experience in questionnaire development. A validated version of the questionnaire was used in this study [16].

The questionnaire included a series of closed- and open-ended questions. The first part of the questionnaire collected demographic details, such as age, gender, educational level attained, monthly income, employment, and marital status. The second part of the questionnaire collected information about diabetes knowledge and awareness through nine closed-ended questions, including questions about risk factors for diabetes. The questionnaire was tested in a pilot study, involving randomly selected 10 participants, to evaluate its design and content. Based on the results, minor modifications were made to the questionnaire. The study employed a simple random sampling approach. Data collection was carried out through social media (WhatsApp©, Facebook©, and Twitter©). Nonresponders were sent reminders and messages encouraging them to complete and return their questionnaires. All questionnaires were completed anonymously. Informed consent was obtained from each participant. All participants were assured of the confidentiality of the information that they provided.

### 2.1. Sample Size

The needed sample size was computed using the Rao soft sample size calculator (http://www.raosoft.com/samplesize.html.) with a 95% confidence level and a 5% margin of error. Because we were unaware of the possible results for each question, we assumed the response distribution for each question would equal 50%. The calculated sample size was 377 individuals. However, we opted to survey at least 430 individuals to increase the dependability of the results.

### 2.2. Data Analysis

Descriptive statistics, including counts and percentages, were calculated for each variable. Statistical Package for Social Sciences version 26.0 (SPSS Inc., Chicago, IL, USA) was used for statistical calculations. Mean ages were calculated. The Chi-square test or Fisher's exact test was used, as appropriate, to assess the association between demographic characteristics and diabetes knowledge questionnaires. A *p* value of <0.05 was considered statistically significant.

## 3. Results

During the study period, a total of 427 subjects were responded to the survey. More than half of the respondents were male 253 (59.3%), while 174 (40.7%) were female, approximately 49% had a university degree and 196 (45.9%) were postgraduates. The mean age of the participants was 25.7 ± 6.2 (mean ± SD). About 33% were employed and 45.2% were students. Most of them were single 230(53.9%) (Table 1).

Among the respondents, 367 (85.9%) had heard of diabetes, whereas 60 (14.1%) had not. Moreover, 366 (85.7%) respondents were aware that it is a lifelong disorder, whereas the remaining 61 (14.3%) were unaware. While 305 (71.4%) participants realized that genetics play a part in disease development, only 147 (34.4%) were unaware of the increased risk. In addition, 236 (55.3%) participants were aware of the disease-associated complications, while 191 (44.7%) were unaware. Overall, 250 (58.5%) believed that diabetes causes foot problems, while 276 (64.6%) were aware that diabetes affects other organs; in contrast, 151 (35.4%) were unaware of this effect. Finally, 352 (82.4%) respondents thought that exercise reduced the risk of diabetes (Table 2).

There was a significant association between the cause of diabetes concerning income and educational status (*p* < 0.05); similarly, there was a significant association among the respondents regarding the question about diabetes-associated complications with age, education, and family income (*p* < 0.05). Results also revealed that there was an association between male and female, educational levels concerning diabetes knowledge (*p* < 0.05). The study parameters related to diabetes knowledge and demographics are given in Table 3.

**Figure 1:**
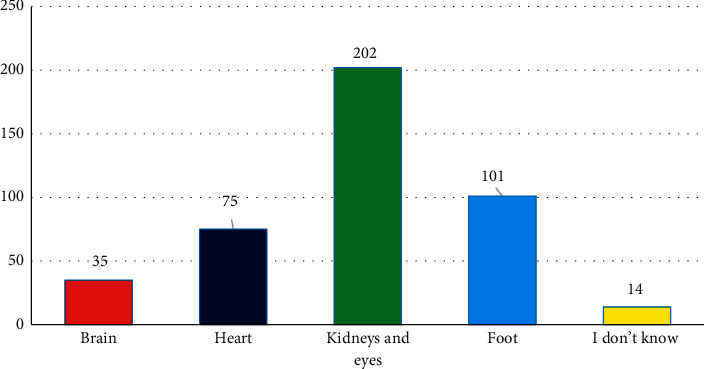
Organs affected by diabetes.

**Figure 2:**
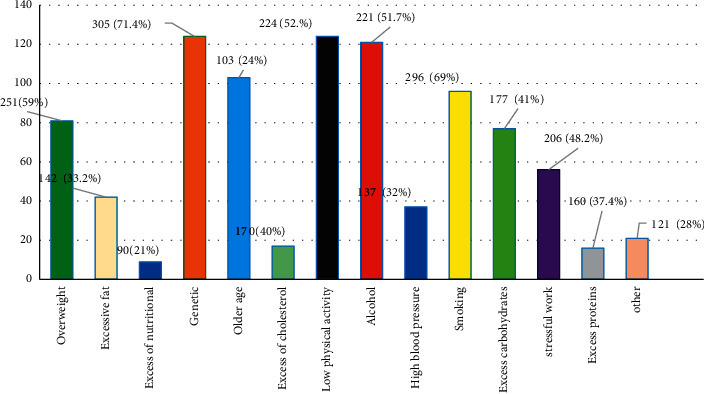
Risk factors associated with diabetes.

When asked which organ was most commonly affected by high sugar levels, 202 (47.3%) of the respondents name kidneys and eyes as affected by diabetes, followed by 101 (23.6%) naming the foot, and 75 (17.5%) naming the heart (Figure 1). Regarding risk factors for diabetes, the majority of the respondents of 305 (72%) said genetic factors, 224 (52%) said lack of physical activity, and 296 (69%) said smoking were the main risk factors for diabetes, while 221 (51.7%), 251 (59%), and 206 (48.2%) named alcohol use, being overweight, and stressful mental work, respectively (Figure 2).

## 4. Discussion

Previous studies have shown that the effective management of blood glucose levels was strongly connected to adequate awareness and knowledge of diabetes; in addition, previous reports have also shown a correlation between diabetes mellitus knowledge and hemoglobin A1C level [17–19]. The present study demonstrated acceptable levels of diabetes awareness and knowledge in the population resident in the state of Telangana, comparable to previous findings from international and national research [20, 21]. This study highlighted several important observations. Overall, the majority (86%) of the study participants were aware of a condition called “diabetes,” and a large percentage knew it to be a lifelong disease; nevertheless, associated complications and affected organs were known to few respondents. Previous studies have suggested that patients with diabetes or a family history of diabetes are likely to have a high level of knowledge about the disease through either their physician or other resources. In our study, the majority of participants correctly identified risk factors for diabetes. This finding shows that regardless of the diabetes status or family history, our study participants had adequate knowledge regarding diabetes. This might be due to the nature of the study sample background of the participants and the type of study instrument used.

A previous study in India found that 90% of the general population was aware of diabetes and its complications [22], which was a considerably higher proportion than that in our study. This discrepancy might be explained by the previous study using a large sample size as well as study design used, whereas our tool was a group of senior researchers-constructed. Similarly, a study by Saleh et al. reported that 82% of the participants were aware of diabetes, which was a smaller proportion than that in our study [20]. However, a study by Deepa et al. involving urban and rural areas of the regions of Chandigarh, Tamil Nadu, Jharkhand, and Maharashtra revealed that only 43.2% of 14,274 respondents had heard about diabetes. This study also reported that urban residents had higher awareness rates (58.4%) than did rural residents (36.8%). These results were inconsistent with our study results. Concurrently, our study found that 61% of the participants were aware of diabetes affecting other organs, compared to 72.7% who reported as having this awareness in a previous study [10].

The prevalence of diabetes is high among the Indian population [23], surprisingly India has got the second-highest position in the prevalence of high rates of diabetes after China [23–27]. Raising awareness of the disease, its causes, treatment, and associated complications is the first step toward disease prevention, which could be achieved through a patient education program, including education on a healthy lifestyle. Few limitations exist in our study. First, the design of the research was an online-based cross-sectional self-reported survey, potentially rendering our results as less reliable. However, because the survey was anonymous and completely voluntary, one can assume that diabetes clinical status was reliably captured. Second, the study was conducted on a single state of the individuals; hence, results cannot be generalized to all public in India. Third, despite assessing the knowledge and awareness of diabetes, the level of knowledge about types of diabetes was not evaluated among the individuals.

## 5. Conclusion

Diabetes poses the main health challenge from both a public perspective and economically all over the world. However, awareness of diabetes is vital for good health and healthy living of individuals. Our findings suggest that awareness and knowledge are inadequate of diabetes, concerning several items in the study including its complications and effects of diabetes on other organs. The need to raise the level of awareness of diabetes among the Indian population is essentially warranted.

## Figures and Tables

**Table 1:** Demographic characteristics of the survey respondents(*n* = 427).

Characteristic	Family history of diabetes		Total
	Yes *n* (%)	No *n* (%)	
*Gender*			
Male	96 (57.5)	157 (60.4)	253 (59.3)
Female	71 (42.5)	103 (39.6)	174 (40.7)

Age (in years) (mean ± std)	25.7 ± 6.2		

*Educational level*			
High school	—	22 (8.5)	22 (5.2)
University degree	77 (46.1)	132 (50.8)	209 (48.9)
Postgraduation	90 (53.9)	106 (40.8)	196 (45.9)

*Employment status*			
Employed	44 (26.3)	96 (36.9)	140 (32.8)
Students	102 (61.1)	91 (35)	193 (45.2)
Business	12 (7.2)	22 (8.5)	34 (8)
Unemployed	9 (5.4)	51 (19.6)	60 (14.1)

*Family Income*			
2963–4893	17 (10.2)	31 (11.9)	48 (11.2)
4894–7322	36 (21.6)	30 (11.5)	66 (15.5)
7323–9787	18 (10.8)	30 (11.5)	48 (11.2)
More than 19575	96 (57.5)	169 (65)	265 (62.1)

*Marital status*			
Married	73 (43.7)	124 (47.7)	197 (46.1)
Single	94 (56.3)	136 (52.3)	230 (53.9)

**Table 2:** Diabetes awareness among the survey participants (*n* = 427).

Variables	Frequency (*n*)	Percentile (%)
*Did you hear about a condition called diabetes?*		
Yes	367	85.9
No	60	14.1

*Do you know it is a lifelong chronic disease?*		
Yes	366	85.7
No	61	14.3

*Do you think diabetes is a genetic disease?*		
Yes	305	71.4
No	122	28.6

*Do you know what happens if you have high sugar levels* ^*∗*^ *?*		
Yes	272	63.7
No	147	34.4

*Do you know of any diabetes-associated complications?*		
Yes	236	55.3
No	191	44.7

*Do you know diabetes can lead to foot problems?*		
Yes	250	58.5
No	177	41.5

*Do you think diabetes can affect other organs in the body?*		
Yes	276	64.6
No	151	35.4

*Do you think the risk of diabetes reduces with exercise?*		
Yes	352	82.4
No	75	17.6

**Table 3:** Diabetes knowledge parameters and their association with demographics.

Parameters	Yes *n*(%)	No *n*(%)	*p*-value
*Do you think diabetes is a genetic disease?*			
Gender	178 (70.4)	75 (29.6)	
Male female	127 (73)	47 (27)	0.554

*Educational level*			
High school	22 (100)	--	
University degree	127 (60.8)	82 (39.2)	0.0001^*∗*^
Postgraduation	156 (79.6)	40 (20.4)	

*Family Income*			
2963–4893	48 (15.7)	--	
4894–7322	35 (53)	31 (47)	
7323–9787	31 (64.6)	17 (35.4)	0.0001
More than 19575	191 (72.1)	74 (27.9)	

*Do you know of any diabetes-associated complications?*			
Gender	121 (47.8)	132 (52.2)	
Male female	115 (66.1)	59 (33.9)	0.0001^*∗*^

*Educational level*			
High school	8 (36.4)	14 (63.6)	
University degree	110 (52.6)	99 (47.4)	0.058^*∗*^
Postgraduation	118 (60.2)	78 (39.8)	

*Family Income*			
2963–4893	20 (41.7)	28 (58.3)	
4894–7322	62 (93.9)	4 (6.1)	0.0001
7323–9787	18 (37.5)	30 (62.5)	
More than 19575	136 (51.3)	129 (48.7)	

*Do you know diabetes can lead to foot problems?*			
Gender			
Male	135 (53.4)	118 (46.6)	
Female	115 (66.1)	59 (33.9)	0.009

*Educational level*			
High school	8 (36.4)	14 (63.6)	0.0001
University degree	98 (46.9)	111 (53.1)	
Postgraduation	144 (73.5)	52 (26.5)	

*Family Income*			
2963–4893	28 (58.3)	20 (41.7)	0.791
4894–7322	42 (63.6)	24 (36.4)	
7323–9787	29 (60.4)	19 (39.6)	
More than 19575	151 (57)	114 (43)	

## Data Availability

The datasets used to support the findings of this study are available from the corresponding author on reasonable request (wali@ksu.edu.sa).
